# Chitosan oligosaccharide (GO2KA1) improves postprandial glycemic response in subjects with impaired glucose tolerance and impaired fasting glucose and in healthy subjects: a crossover, randomized controlled trial

**DOI:** 10.1038/s41387-019-0099-4

**Published:** 2019-11-04

**Authors:** Sarang Jeong, Jung Min Cho, Young-In Kwon, Seong-Chul Kim, Dong Yeob Shin, Jong Ho Lee

**Affiliations:** 1 National Leading Research Laboratory of Clinical Nutrigenetics/Nutrigenomics, Department of Food and Nutrition, College of Human Ecology, Yonsei University, Seoul, Korea; 2 Department of Food and Nutrition, Hannam University, Daejeon, 305-811 Korea; 3 Institute of Functional Foods, KunpoongBio Co. Ltd., Jeju, 63010 Korea; 4 Department of Internal Medicine, Severance Hospital, Division of Endocrinology and Metabolism, Yonsei University College of Medicine, 50-1 Yonsei-ro, Seodaemun-gu, Seoul, 03722 Korea

**Keywords:** Nutritional supplements, Nutrition

## Abstract

**Background:**

The antidiabetic and hypoglycemic effects of chitosan have been reported in previous studies. We have previously shown that chitosan oligosaccharide reduces postprandial blood glucose levels in vivo. We conducted a short-term crossover study to support the results of the previous study.

**Methods:**

The study was a randomized, double-blind, controlled crossover trial completed at one clinical research site. Subjects with impaired glucose tolerance and impaired fasting glucose and healthy subjects were randomly assigned to consume one of two different experimental test capsules that differed in only the sample source (GO2KA1 vs placebo), and all subjects were instructed to consume the 75 g sucrose within 15 min. After a 7-day interval, the subjects consumed the other capsules that were not consumed on the first day. We assessed blood glucose levels using a 2-h oral sucrose tolerance test. The study was registered at clinicaltrials.gov (NCT03650023).

**Results:**

The test group showed significantly lower blood glucose levels at 60 min (*p* = 0.010) and postprandial blood glucose areas under the curve (*p* = 0.012). The change in blood glucose levels at 60 min was significantly lower in the test group than in the placebo group (*p* = 0.017).

**Conclusions:**

Based on the results of this study, the consumption of chitosan oligosaccharide (GO2KA1) supplements with a meal can effectively reduce postprandial blood glucose levels, which is relevant to the prevention of diabetes.

## Introduction

Healthy dietary and lifestyle changes can reduce the risk of chronic noncommunicable diseases (NCDs), including obesity, diabetes mellitus (DM), cardiovascular disease (CVD), hypertension, stroke, and certain types of cancer^1^. In particular, diabetes is one of the major NCDs whose prevalence is steadily increasing globally and that has become a major challenge to public health and health care systems. Diabetes is characterized by hyperglycemia resulting from defects in insulin secretion, insulin action, or both. According to the World Health Organization (WHO), the global prevalence of diabetes among adults over 18 years of age rose from 4.7% in 1980 to 8.5% in 2014. In 2016, diabetes was the direct cause of 1.6 million deaths, and in 2012, high blood glucose was the cause of another 2.2 million deaths^2^. Postprandial glycemic control has been shown to play an important role in overall blood glucose management and the delay of its metabolic complications^3^.

According to the clinical diagnosis guidelines in the ‘Prevention and Control of Noncommunicable Diseases: Guidelines for primary health care in low-resource settings’ of the WHO, DM can be diagnosed based on 2-h plasma glucose levels from the 75 g oral glucose tolerance test (OGTT) that are ≥7.0 mmol/L (126 mg/dL) or ≥11.1 mmol/L (200 mg/dL). Impaired glucose tolerance (IGT) can be diagnosed based on 2-h plasma glucose levels from the OGTT in the range of 7.8–11.1 mmol/L (140–200 mg/dL) with a fasting glucose level <7.0 mmol/L (<126 mg/dL). Impaired fasting glucose (IFG) can be diagnosed based on 2-h plasma glucose levels from the OGTT that are <7.8 mmol/L (140 mg/dL) with fasting blood glucose levels in the range of 6.1 to 6.9 mmol/L (110 mg/dL to 125 mg/dL)^4^. In addition, according to the ‘Diabetes Care’ guidelines of the American Diabetes Association (ADA), the same thresholds used by the WHO are applied for IGT but a lower cutoff value is used for IFG (FPG 5·6–6·9 mmol/L)^5^. IGT, IFG and healthy glucose statuses are also called ‘prediabetes’ or ‘nondiabetes’. Thus, we defined prediabetes as IGT and IFG and nondiabetes as healthy. Prediabetes is a high-risk state for diabetes development^6^. Moreover, uncontrolled diabetes often leads to biochemical imbalances that can cause acute life-threatening events, such as diabetic ketoacidosis and hyperosmolar (nonketotic) coma. Furthermore, complications of diabetes include heart disease, stroke, hypertension, blindness, eye problems, kidney disease, nervous system disease, nontraumatic lower-limb amputations, and dental disease^7^. Therefore, controlling blood glucose is an important strategy to prevent diabetes-related health complications.

In terms of functional foods, research is also underway regarding the utilization of ingredients from bioactive compounds and natural substances. The Korea Ministry of Food and Drug Safety recognized materials that maintain or improve health by improving human function and exerting positive biological effects as functional ingredients^8^. Thus, in recent decades, functional foods, as an important factor for health, have received more interest from researchers^9–11^.

Normal glucoregulation is maintained by an intricate interaction between pancreatic β-cells (insulin/amylin), pancreatic α-cells (glucagon), and associated organs (e.g., intestines, liver, skeletal muscle, adipose tissue)^12^, i.e., a decrease insulin secretion stimulates glucagon secretion during hypoglycemia^13^. In other words, the insulin and glucagon are involved in the blood glucose adjustment or euglycemia state. In pancreatic β-cells, the glucose metabolism signals insulin secretion by altering the cellular array of messenger molecules (e.g., food intake, digestion, and glucose consumption resulting from exercise)^14^. Whereas, glucagon that is secreted from pancreatic α-cells, which also has a central role in glucose homeostasis, is produced in response to low glucose levels or hypoglycemia and acts to increase glucose levels (hyperglycemia) by accelerating glycogenolysis and promoting gluconeogenesis^15^. And, at the same time, inhibits glycogengenesis of glycogen synthesis in the liver and suppress glycolysis^16^. Furthermore, as with insulin, glucagon influence blood glucose dose–response curve is sigmoidal^17^.

Chitosan is present in the endoskeletons of mollusks (e.g., squid and octopus)^18^ and in the cell walls of mushrooms^19^. Chitosan consists of 2-acet-amido-2-desoxy-β-d-glucopyranose and deacetylated 2-amino-2-deoxy-β-d-glucopyranose monomers, and the amount of deacetylated monomers exceeds the amount of acetylated ones^20^. Chitosan oligosaccharides are the hydrolyzed products of chitin, which is abundant in the exoskeleton of crustaceans (e.g., crab, crawfish, shrimps) and the cell walls of fungi^21^. Chitosan is a linear polysaccharide composed of randomly distributed-(1-4)-linked d-glucosamine and *N*-acetyl-d-glucosamine^22,23^. In addition, chitosan has been reported to have other biological properties, such as the reduction of total cholesterol^24^ and the inhibition of intestinal absorption of dietary fat^25^.

The antidiabetic and hypoglycemic effects of chitosan have been reported in previous studies^26,27^. We have previously shown that chitosan oligosaccharide reduces postprandial blood levels in vivo^28^. In addition, Jo SH et al.^29^ reported that GO2KA1 exerted glucose-lowering effects in normal and prediabetic individuals in a rat model and in vitro.

Our previous study showed that chitosan oligosaccharide (GO2KA1) supplementation was more effective for the treatment of prediabetes than roasted barley meal powder^30^. However, we need to ensure the effect of chitosan oligosaccharide on postprandial blood glucose regulation. Egg white powder does not affect postprandial blood glucose compared with the effect of roasted barley meal powder. Therefore, we chose egg white powder as the treatment for the control group. In addition, blood glucose control is an important factor in the prevention of diabetes^31,32^, and since previous research has analyzed insulin and HbA1c, the current study was conducted as a support study, and blood glucose levels were used as the primary endpoint.

The current study was conducted to support previous studies that evaluated the impact of GO2KA1 on blood glucose control in Korean populations with IGT, IFG, and a healthy glucose status.

## Methods

### Study subjects

For the study sample size determination, we determined the size of in the sample for the study by referring to other study designs, such as our previous study design^25–27^. Our study is exploratory in nature. Thus, it is better to determine the sample size for the study by referring to other studies rather than using an equation. Finally, we decided on 40 study subjects, considering a dropout of 25%.

**Fig. 1:**
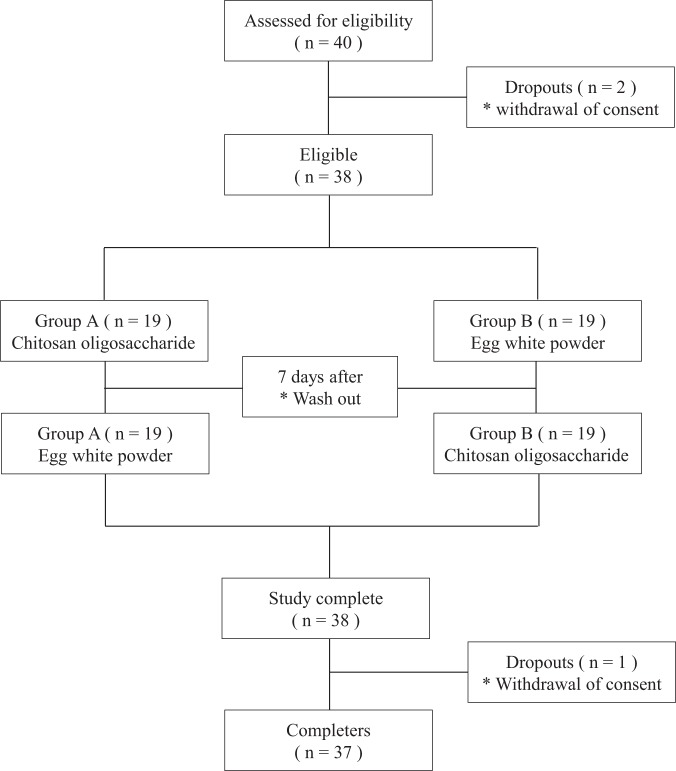
Flow of the study

The study was a randomized, double-blind, controlled crossover trial completed at one clinical research site. The study subjects included were between the ages of 20 and 75 years and signed a consent form. A total of 40 subjects with normal fasting blood glucose and glucose tolerance (OGTT ≤ 140 mg/dL with fasting blood glucose ≤ 100 mg/dL), impaired fasting glucose (IFG; OGTT < 140 mg/dL with fasting blood glucose from 110–125 mg/dL), and impaired glucose tolerance (IGT; OGTT 140–200 mg/dL with fasting glucose <126 mg/dL) except diabetic subjects determined by an OGTT at screening were recruited from the Clinical Nutrigenetics/Nutrigenomics Laboratory at Yonsei University from 14 May 14 ~ 4 June 2018. Thirty-seven subjects were included in the final analysis since three subjects did not complete all test requirements, including the 2-h oral sucrose tolerance test (OSTT). And the subjects are considered a group. The subjects had no history/presence of DM, cerebrovascular diseases, heart disease, hypertension, kidney disease, liver disease, gastrointestinal disease, alcoholism, drug addiction, or any other acute or chronic disease requiring treatment. The subjects did not use any medication affecting glucose control for 1 month before the screening. The exclusion criteria were as follows: pregnancy or breastfeeding, history of cancer, cancer surgery and hospitalization (Fig. 1).

The aims of this study were carefully explained to all subjects, and written consent was obtained prior to participation. The Institutional Review Board (IRB) of Yonsei University (IRB No. 7001988-201807-HR-369-04) and the Severance Hospital approved the study protocol, which complied with the Declaration of Helsinki. This trial is registered under the clinicaltrials.gov trial registration code: NCT03650023 (https://register.clinicaltrials.gov/).

### Anthropometric assessments and body composition measurements

The body weight and height of the subjects were measured without heavy clothing or shoes in the morning. The body mass index (BMI, Inbody370; Biospace, Cheonan, Korea) was calculated as kilograms per square meter (kg/m^2^). Waist and hip circumference (measured directly on the skin) was measured at the umbilical level after normal expiration using a plastic measuring tape with measurements to the nearest 0.1 cm while the participant was standing in an upright position. Systolic and diastolic blood pressures (BPs) were assessed in a sitting position with the arms supported precisely at the atrium level after a resting period (20 min). BP was measured twice on the arm using an automatic BP monitor (FT-200S; Jawon Medical, Gyeongsan, Korea); the two or three measurements that were taken were then averaged.

### Application of OSTT

The OSTT is known to induce a greater increase in insulin levels than the OGTT^33,34^. Therefore, this characteristic might indicate that the OSTT is a more sensitive tolerance test than the OGTT; in the present study, we chose the OSTT method. In addition, our previous published study reported that low molecular weight (< 1000 Da) chitosan oligosaccharide (GO2KA1) significantly reduced postprandial blood glucose levels via the inhibition of α-glucosidase in both normal and diabetic animals using an OSTT^29,35^. Thus, we designed this study to reconfirm the glucose-level-lowering effect using the same OSTT method in the current human clinical trial with prediabetic subjects.

### 2-h OSTT

For the crossover trial, subjects were instructed to consume 75 g sucrose and the chitosan oligosaccharide (GO2KA1) or the placebo (egg white powder), which were provided in the form of two capsules. Each capsule contained 250 mg of each test sample: GO2KA1 (KUNPOONG BIO CO., LTD, Korea) and placebo. Chitosan oligosaccharide (GO2KA1) contains 365 kcal per 100 g. The egg white powder used in this study was composed of carbohydrates (9%), protein (91%), and 382 kcal per 100 g. The egg white powder was individually approved by the Ministry of Food and Drug Safety. On the first day of the experiment, subjects were randomly assigned to consume one of the two types of experimental test capsules that differed in only the sample source (chitosan oligosaccharide vs egg white powder), and all subjects were instructed to consume the 75 g sucrose within 15 min. After a 7-day interval, the subjects consumed the other capsules that were not consumed on the first day. Blood samples were collected after an overnight fast of at least 12-h. Venous blood specimens were collected in serum tubes. On both days, the OSTT was performed via a 75 g sucrose challenge, and serum glucose concentrations were determined at 0, 30, 60, 90, and 120 min. The blood samples were centrifuged (3000 rpm, 10 min, 10 °C) to obtain serum samples, which were then stored at – 80 °C.

### Blood collection

For the crossover trial, blood samples were collected after an overnight fast of at least 12-h. Venous blood specimens were collected in ethylenediaminetetraacetic acid-treated whole-blood tubes and serum tubes (BD Vacutainer; Becton, Dickinson and Company, Franklin Lakes, NJ, USA) in a fasting state (0 min) and at 30, 60, 90, and 120 min after consuming the test samples and the 75 g sucrose. The blood samples in the collection tubes were immediately placed on ice until they arrived at the analytical laboratory (within 1–3 h). The blood samples were centrifuged to obtain plasma and serum samples, which were then stored at – 80 °C.

### Assessment of nutrient intake and physical activity

The study subjects were interviewed at each visit to obtain information about their nutrient intake and physical activity. The study subjects maintained their usual diet and physical activity habits during the 2-week intervention period. Dietary intake was assessed using a semiquantitative food frequency questionnaire and a 24-h recall method. Nutrient intake was determined and calculated based on the 3-day food records using the Computer Aided Nutritional Analysis Program (CAN-pro 3.0, Korean Nutrition Society, Seoul, Korea). Total energy expenditure (TEE) (kcal per day) was calculated based on the activity patterns of the study subjects, such as the basal metabolic rate (BMR), 24-h physical activity, and the specific dynamic actions of food. The BMR for each subject was calculated with the Harris–Benedict equation.

### Statistical analysis

The statistical analyses were performed using SPSS version 24.0 software (IBM/SPSS, Chicago, IL, USA). We examined whether each variable was normally distributed before statistical testing, and logarithmic transformation was performed on the skewed variables. For descriptive purposes, mean values are presented for the untransformed variables. The results are expressed as the means ± standard errors. For the comparison of categorical variables, a chi-squared test was conducted. Paired *t* tests were used to compare the parameters collected from the test group and those collected from the placebo group at each visit. The areas under the curve (AUCs; mg/dL × h) were calculated by using the trapezium rule to represent the total response. The one-way analysis of variance and Bonferroni methods were used within each group. Two-tailed *p* values < 0.05 were considered to indicate statistical significance.

## Results

The recruitment and follow-up period was from 14 May 2018 ~ 4 June 2018. The dropout rate was lower than 25%. Some subjects were excluded for personal reasons (*n* = 3). Thus, 37 subjects completed the crossover trial.

### Clinical characteristics and anthropometric assessments

The basic clinical characteristics and anthropometric assessments of the 37 subjects are shown in Table 1. There was no significant difference between the groups in terms of basic clinical characteristics: (i.e., weight, BMI, waist and hip circumference, waist-hip ratio, systolic and diastolic BP, heart rate).

**Table 1:** Characteristics of the study subjects

	Total subjects (*n* = 37)	
	Visit 1	Visit 2
Age (years)	28.6 ± 1.49	
Male/female (*n*, %)	18 (48.6)/19 (51.4)	
Current smoker (*n*, %)	4 (10.8)	
Current drinker (*n*, %)	29 (78.4)	
Height (cm)	168.0 ± 1.48	
Weight (kg)	64.2 ± 1.85	64.1 ± 1.87
BMI (kg/m^2^)	22.6 ± 0.45	22.6 ± 0.46
Waist (cm)	84.9 ± 1.02	84.9 ± 1.05
Hip (cm)	96.7 ± 1.22	97.4 ± 0.96
Waist-hip ratio	0.88 ± 0.01	0.87 ± 0.01
Systolic BP (mmHg)	113.3 ± 1.76	112.0 ± 1.80
Diastolic BP (mmHg)	67.3 ± 1.12	66.6 ± 1.01
Heart rate (bpm)	71.7 ± 1.39	71.1 ± 1.53
Glucose (mg/dL)	95.1 ± 0.89	93.7 ± 0.98

Mean ± SE. There were no significant differences between visit 1 and visit 2

### Laboratory measurements regarding the safety aspects

During the consumption of the GO2KA1 or the placebo (egg white powder) capsules, no obvious side effects were reported. The basic laboratory measurements of the 37 subjects are shown in Table 2. The laboratory measurement results were within the normal range. There was no significant difference between the groups in terms of basic laboratory measurements: (i.e., glutamic oxaloacetic transaminase, glutamic pyruvic transaminase, serum albumin, blood urea nitrogen, creatinine, white blood cells, red blood cells, hemoglobin, hematocrit, platelets).

**Table 2:** Laboratory measurements, daily food intake and total energy expenditure

	Total subjects (*n* = 37)	
	Screening	
*Laboratory tests*		
GOT (IU/L)	19.22 ± 0.61	
GPT (IU/L)	16.86 ± 1.13	
Serum albumin (g/dL)	4.85 ± 0.04	
BUN (mg/dL)	13.14 ± 0.55	
Creatinine (mg/dL)	0.83 ± 0.03	
	**Placebo (*n*** **=** **37)**	**GO2KA1 (*n*** **=** **37)**
White blood cells (× 10^3^/μL)	5.78 ± 0.25	5.79 ± 0.27
Red blood cells (× 10^6^/mm^3^)	5.11 ± 0.10	5.01 ± 0.10
Hemoglobin (g/dL)	14.15 ± 0.31	13.89 ± 0.31
Hematocrit (%)	45.65 ± 1.01	44.85 ± 1.01
Platelets (× 10^3^/mm^3^)	226.78 ± 7.98	230.81 ± 8.21
*Daily food intake and total energy expenditure*		
Basal metabolic rate (kcal/d)	1576.0 ± 82.20	1577.5 ± 82.00
Total energy expenditure (kcal/d)	2296.6 ± 54.55	2295.6 ± 52.79
Estimated energy intake (kcal/d)	2296.0 ± 47.51	2302.3 ± 48.24
Carbohydrates (%)	62.0 ± 0.24	61.7 ± 0.15
Protein (%)	15.7 ± 0.26	15.8 ± 0.06
Fat (%)	22.5 ± 0.15	22.6 ± 0.19
Cholesterol (mg)	179.6 ± 0.40	179.7 ± 0.47

Mean ± SE. There were no significant differences between the placebo and GO2KA1 groups

### Nutrient intake and physical activity

The BMR (kcal/d; 1577.25 ± 82.00 and 1576.0 ± 82.20), TEE (kcal/d; 2295.6 ± 52.79 and 2296.6 ± 54.55), estimated energy intake (kcal/d; 2302.3 ± 48.024 and 2296.0 ± 47.51), percentages of macronutrient intakes (CHO: PRO: FAT = 61.7: 15.8: 22.6 and 62.0: 15.7: 22.5), and cholesterol (mg; 179.7 ± 0.47 and 179.6 ± 0.40) of the chitosan oligosaccharide (GO2KA1), and placebo groups did not differ significantly (Table 2).

### Serum concentrations of blood glucose and the AUCs

**Fig. 2:**
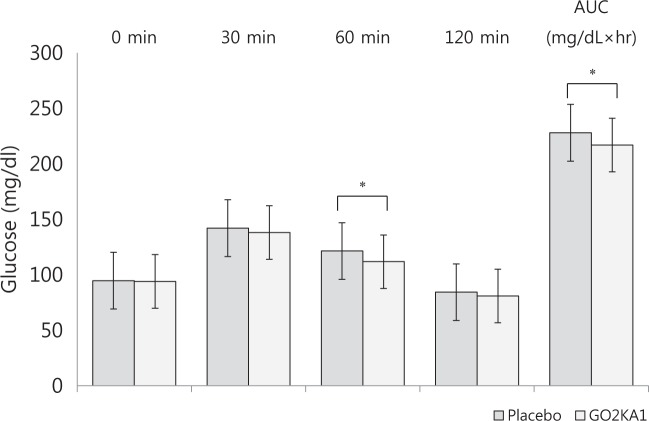
Effects of GO2KA1 on blood glucose in the oral sucrose tolerance test (OSTT). Mean ± SE. *P < 0.05 derived from paired *t* tests between the placebo and GO2KA1 groups at 0 min, 30 min, 60 min, and 120 min and the areas under the curve (AUCs)

Table 3 shows the serum concentrations of blood glucose during the OSTT. The serum concentrations of blood glucose indicate that the blood glucose level peaked at 30 min and returned to baseline by 2-h after the OSTT. The mean blood glucose levels were significantly lower 60 min (*p* *=* 0.010) after the consumption of the GO2KA1 capsules than after the consumption of the placebo capsules. The postprandial blood glucose AUC (mg/dL × h) was significantly lower (*p* = 0.012) after the consumption of the GO2KA1 capsules than after the consumption of the placebo capsules. Although not significant, the mean postprandial blood glucose levels 30 and 120 min after consuming the GO2KA1 capsules tended to be lower than the levels after consuming the placebo (Fig. 2).

**Table 3:** Results of the oral sucrose tolerance test (OSTT) and the areas under the curve (AUCs)

	Placebo (*n* = 37)	GO2KA1 (*n* = 37)	*p*
*Glucose (mg/dL)* ^*∮*^			
0 min	94.7 ± 0.97^*c*^	94.0 ± 0.90^*c*^	0.415
30 min	142.0 ± 4.31^*a*^	138.1 ± 3.50^*a*^	0.292
60 min	121.4 ± 4.77^*b*^	111.8 ± 4.00^*b*^	**0.010**
120 min	84.3 ± 2.65^*d*^	80.9 ± 2.44^*d*^	0.133
AUC (mg/dL × h)	227.9 ± 5.77	216.9 ± 4.80	**0.012**

Mean ± SE. ^*∮*^tested following logarithmic transformation. *p* values derived from a paired *t* test between the placebo and GO2KA1 groups. All alphabetical *p* values were derived from a one-way ANOVA with a Bonferroni correction within each group; no significant differences are marked with the same letter, and significant differences are marked with a different letter

*AUC* area under the curve

### The change in the concentration of glucose in the blood and the concentrations of glucose at each minute after a 12-h fast (0 min)

**Fig. 3:**
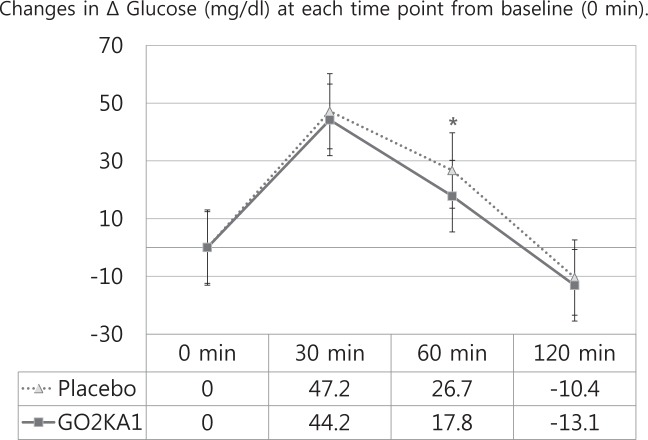
Mean ± SE. *P < 0.05 derived from paired *t* tests between the placebo and GO2KA1 groups at 0 min, 30 min, 60 min, and 120 min. Blood glucose change values from the initial value (0 min) at 0 min, 30 min, 60 min, and 120 min

Table 3 shows the changes in the serum concentrations of blood glucose from the initial values (Δ) after the OSTT (placebo vs GO2KA1). The Δ glucose (mg/dL) at 60 min was significantly lower (*p* = 0.017) after the consumption of the GO2KA1 capsules than after the consumption of the placebo capsules (Fig. 3).

## Discussion

The randomized, double-blind, crossover trial evaluated the effect of GO2KA1 on glucose control among subjects with IGT, IFG, and a healthy glucose status. We found that compared with a placebo, the GO2KA1 supplement improved the serum glucose levels at 60 min and the AUCs.

Several previous studies have investigated the beneficial effects of chitosan oligosaccharide on glucose control. Chitosan oligosaccharide was shown to act as an antidiabetic agent because it increases glucose tolerance and insulin secretion and decreases triacylglycerides^36^. In one study, low molecular weight chitosan was shown to prevent the progression of low-dose streptozotocin-induced slowly progressive DM in mice^27^. Our previous study was a randomized, double-blind, placebo-controlled 12-week intervention trial that reported that low molecular weight chitosan oligosaccharide (GO2KA1) effectively reduced glucose levels at 30 and 60 min in subjects with prediabetes^30^. However, there are few intervention studies about chitosan oligosaccharide and glucose control in humans. In addition, we wanted to verify the evidence supporting this result. Therefore, we conducted a support study to support the findings of a previous study by using egg white powder as a placebo as it does not affect postprandial blood glucose. According to the criteria applied, we were classified by IGT, IFG, and healthy glucose status through OGTT. However, they were not clearly diabetic. Thus, IGT, IFG, and a healthy glucose status were considered as one of prediabetes or healthy subjects.

The chitosan oligosaccharide (GO2KA1) capsule contained enzymatically digested low molecular weight chitosan oligosaccharide (500 mg per dose). The placebo capsule contained egg whites (99.7%), fresh yeast (0.1%), and citric acid (0.2%). In our previous study, we used roasted barley powder as a placebo. That placebo capsule contained 100% roasted barley powder, which was purchased from a local market in Jeju and was prepared as a powder using a grinder (JEIL Industry) at room temperature (25 °C)^30^. It is important to note that the glycemic index of barley is fifty. The egg whites used in this study contained carbohydrates (9%), protein (91%), and 382 kcal per 100 g. The glycemic index of egg whites (500 mg egg white powder) is zero^37^ since small amounts of carbohydrate have little effect on postprandial blood glucose. Clearly, the glucose index of egg whites used as placebo is lower than that of barley. Thus, in this study, egg whites were chosen as a placebo to support the findings of the previous study regarding the effect of chitosan oligosaccharide (GO2KA1).

According to the ADA Diabetes Care guidelines from 2001, in nondiabetic individuals, blood glucose concentrations peak 60 min after meals (at this time, the level is a little higher than 140 mg/dL) and return to preprandial levels within 2–3 h^38^. However, in the context of DM, IGT, and IFG, blood glucose is difficult to control at all times, in addition to the 60 min after meals. Therefore, it is meaningful to reduce blood glucose levels or the overall increase in blood glucose levels 60 min after meals. In this study, we investigated the effect of chitosan oligosaccharide (GO2KA1) on postprandial blood glucose control. Chitosan oligosaccharide (GO2KA1), when consumed in the form of a capsule of powder, suppresses the postprandial increase in serum levels of blood glucose compared with the effect of egg whites. The blood glucose levels of the GO2KA1 group at 60 min were significantly lower than the levels of the placebo group. In addition, the postprandial blood glucose AUCs (mg/dL × h) and Δ glucose (mg/dL) of the GO2KA1 group at 60 min were significantly lower than those of the placebo group. The current study revealed that blood glucose peaks at 30 min. This result was thought to be because the study subjects were not diabetes patients^39,40^.

This study utilized a crossover design that aimed to support the results of our previous study. However, the limitations of this study include the small number of subjects and the shortcomings of the crossover trial design, such as the carry-over effect^41^. To avoid carry-over effects, we used a 7-day washout period. Because the half-lives of blood glucose and endogenous insulin are approximately 11 min^42^ and 4–6 min^43^, respectively, we considered 7 days to be sufficient. Despite these limitations, this study shows that the GO2KA1 supplement improved the postprandial blood glucose levels and AUCs, compared with the effect of the placebo, in individuals with normal blood glucose, IFG, and IGT. Because the half-life of HbA1c is approximately 44 days^44^, we thought that in the current short-term study, it was unsuitable for the primary endpoint. Although we did not measure insulin, C-peptide or HbA1c levels as endpoints, previous research has examined these factors. HJ Kim et al.^30^ conducted a study showing the effects of GO2KA1 on subjects with prediabetes, and the finding was that the serum C-peptide level measured at 120 min in the placebo group tended to increase after 12 weeks of the intervention (*P* = 0.046). The change in the serum level of C-peptide at 120 min in the test group tended to be different from that in the placebo group (*P* = 0.069). The plasma level of HbA1c (*P* = 0.023) was reduced in the GO2KA1 intervention group after the 12-week treatment. However, the serum insulin level did not change significantly. The OSTT was selected as an experimental method because it is known to induce a greater increase in insulin levels than the OGTT, but the OGTT was selected as a screening method to diagnose the subjects. To our knowledge, the OGTT criteria are the most accepted selection standard for the diagnosis of IFG and IGT^5^. The mixed use of the OSTT for the experimental method and the OGTT for the inclusion criteria can be a limitation of this study. However, because the OSTT can induce more instant insulin level changes^33,34^ and show more sensitive results, we used the OSTT to obtain clearer results. In addition, chemical structure of sucrose is disaccharides. It consists of one molecule fructose and one molecule glucose^45^. Thus, in humans, sucrose is more like digestion processing than monosaccharides glucose.

Based on the results of this study, the consumption of chitosan oligosaccharide (GO2KA1) supplements with a meal can effectively reduce postprandial blood glucose levels, which is relevant to the management of type 2 diabetes. However, the positive effects in the context of diabetes cannot be confirmed because the study was not conducted with diabetic patients. Thus, further crossover trial studies are required to re-evaluate the effects on the insulin and C-peptide levels, and a larger intervention trial with a larger number of subjects or diabetic patients is needed to confirm the effect of GO2KA1.
